# Prevalence of Health Problems Targeted by the National School-Based Screening Program among Primary School Students in Saudi Arabia, 2019

**DOI:** 10.3390/healthcare9101310

**Published:** 2021-09-30

**Authors:** Manal Matar Al Daajani, Dina Mohammed Al-Habib, Mona Hamed Ibrahim, Nora Abdulrhman Al Shewear, Yahya Mohammad Fagihi, Abrar Abdulazeem Alzaher, Amjad Fawzi Alfaleh, Khaled Ibrahim Alabdulkareem

**Affiliations:** 1 Public Health Department, Health Affairs, Jeddah 22246, Saudi Arabia; 2 General Administration of School Health, Ministry of Health, Riyadh 11176, Saudi Arabia; dalhabib@moh.gov.sa (D.M.A.-H.); ibrahimmd@moh.gov.sa (M.H.I.); alshewearna@moh.gov.sa (N.A.A.S.); ymfagihi@moh.gov.sa (Y.M.F.); ababalzaher@moh.gov.sa (A.A.A.); amfalfaleh@moh.gov.sa (A.F.A.); 3 Department of Community, Environmental and Occupational Medicine, Faculty of Medicine, Zagazig University, Zagazig 44519, Egypt; 4 General Directorate of Medical Consultation, Ministry of Health, Riyadh 11176, Saudi Arabia; 5 Family Medicine Department, College of Medicine, Al Imam Mohammad Ibn Saud Islamic University (IMSIU), Riyadh 11432, Saudi Arabia; khalabdulkarim@moh.gov.sa; 6 Primary Health Care, Assisting Deputyship for Primary Health Care, MOH, Riyadh 11176, Saudi Arabia

**Keywords:** health status, national survey, KSA, school health, students

## Abstract

The Saudi Ministry of Health (MOH) established a National School-Based Screening Program (NSBSP) for health screening of schoolchildren. Students from specific grades were systematically screened for several health problems, including obesity, visual and auditory problems, dental cavities, scoliosis, and attention-deficit/hyperactivity disorder (ADHD). This cross-sectional study aimed to determine the prevalence of these health problems among primary school students based on secondary data obtained from the NSBSP. We included 444,259 screened school children from the first and fourth grades of 50% of the selected schools (both private and public) across the Kingdom of Saudi Arabia (KSA) during the academic year 2018–2019. Among them, the most prevalent health problems identified were dental cavities (38.7%), eye refractory errors (10.9%), and overweight and obesity (10.5%); the less prevalent problems included ADHD (2.81%), auditory problems (0.6%), and scoliosis (0.48%). A greater prevalence of most health problems was observed in girls more than boys. The NSBSP successfully aided the detection of health conditions with high and low prevalence among primary school students in the KSA, and thus, the identification of health problems of specific concern. Implementation of effective school health services for the prevention, early detection, diagnosis, and treatment of these health problems are imperative.

## 1. Introduction

Schools play a very important role in childhood development, education, and psychological and social health, which are fundamental for the quality of life and future of students. Evidence confirms that healthier students are better learners [1]. Health barriers to learning, such as vision and hearing deficits and dental pain, exert adverse effects on learning processes, attendance of the student, academic patterns, and development [2]. Barriers to learning are believed to negatively affect the future financial stability and social life of students. Nearly 80% of learning processes occur through visual tasks, such as writing, reading, and using computers. Untreated visual problems affect students’ ability to read [3], while uncorrected auditory problems are associated with higher rates of dysfunction in speech, language, poor performance on educational tasks, social and emotional difficulties, and lower self-esteem [4]. Dental pain and dental cavities lead to school absences, sleep disturbances, and unaccomplished homework [5]. Several health problems are prevalent in school-age children. The aim of health screening programs is to increase the early detection of health problems and promote prevention of accompanying physical, mental, and social consequences.

The Saudi Ministry of Health (MOH) established the NSBSP in 2018–2019. The program targeted first and fourth grade students of primary schools, first grade students of intermediate schools, and first grade students of secondary schools. This program targeted a set of health problems, including weight-related health problems, visual and auditory problems, dental cavities, smoking, scoliosis, depression, and attention-deficit/hyperactivity disorder (ADHD). One of the objectives of this program was to create a database for recording health problems and to assess the efficacy of appropriate health interventions to prevent these health problems.

ADHD is the most common mental disorder diagnosed in children and usually begins in childhood and becomes prominent in later school years. The global prevalence of ADHD ranges from 2% to 18% [6]. ADHD screening is encouraged in early childhood to promote early diagnosis and symptom management to improve the functioning of children and their families [6]. Scoliosis is an abnormal lateral curvature of the spine, as defined by the American Association of Neurological Surgeons [7]. It is often diagnosed in childhood or early adolescence. Scoliosis screening at early ages is important to detect abnormalities and promptly launch conservative treatment in order to avoid necessitating invasive surgery later in life [7]. Obesity is a global health issue among schoolchildren. In 2016, the World Health Organization (WHO) reported that more than 340 million school-aged children were obese [8]. It is well known that childhood obesity continues into adulthood and it is a risk factor for other several chronic health conditions later in life [9]. Similarly, other diseases and health problems that arise in childhood will affect students’ health and impact their quality of life and future opportunities. This study aimed to determine the prevalence of health problems targeted by the NSBSP among primary school students in the KSA in 2019.

## 2. Materials and Methods

This is a cross-sectional study based on a secondary analysis of data obtained from the NSBSP. This study was conducted by the General Administration of School Health (GASH) during the academic year 2018–2019. Data analysis was performed on students from both private and public primary schools from the 20 health regions of the KSA. We included screening data from the first and fourth grades of primary schools in the KSA. The total number of participants was 444,259, excluding students with incomplete data who were excluded from the analysis (*n* = 3009).

NSBSP is a routine surveillance health program organized and implemented by the GASH at the MOH. Well-trained health teams participated in the screening program. Each health team comprised physicians, nurses, and dentists under the direct supervision of GASH officials. Following examination and screening of students, data obtained during screening were entered into the electronic system of the health screening program. The collected data included sociodemographic characteristics (age, sex, school grade, and region), immunization status, body mass index, visual acuity, dental cavities, hearing problems, scoliosis, and ADHD.

To assess immunization status, health teams inquired about the completion of preschool immunizations as specified by the National Advisory Committee on Immunization. The Saudi immunization schedule includes the oral polio vaccine; measles, mumps and rubella vaccine; varicella; diphtheria tetanus vaccine; and whole-cell pertussis vaccine (or diphtheria, tetanus, and pertussis vaccine for those 7 years or older). Moreover, the health team physician assessed meningococcal vaccine and influenza vaccine by examining the student’s immunization schedule card [10].

Health teams nurses calculated the body mass index (BMI) after measuring the weight and height of each student. BMI data were plotted on Saudi’s sex-specific percentile charts and categorized into four groups: underweight (less than 5th percentile); normal weight (between 5th percentile and 85th percentile); overweight (between 85th and 95th percentile); and obese (more than 95th percentile) [11]. Health teams examined visual acuity and tested students near vision using Snellen charts. Students who scored ≤6/12 were considered to be myopic. Dentists assessed the students for the presence of dental cavities and reported them in the electronic records. When the teacher suspected that a student had a hearing problem, the health teams examined student’s hearing using an audiometer. The present analysis includes hearing examination data of the first-grade students only. We considered the normal hearing range 1000–4000 Hz at an intensity of 25 dB [12]. Health team physicians assessed scoliosis in fourth grade students using Adam’s forward bending test [7]. ADHD was assessed in suspected first grade students using the Vanderbilt ADHD Rating Scale based on assessment forms received from parents and teachers.

### 2.1. Statistical Analysis

Data analysis was performed using SPSS version 21 (IBM, Armonk, NY, USA). The data were cleaned for incomplete or missing variables. Qualitative data are presented as frequencies and percentages, and quantitative data are presented as mean ± standard deviation (SD). Pearson’s chi-square test was used to analyze the categorical variables. A two-sided significance test was used, with a *p* value < 0.05.

### 2.2. Ethical Considerations

Approval to conduct this study was obtained from the Institutional Review Board (IRB) of the MOH prior to study execution.

## 3. Results

The total number of students enrolled in this study was 444,259, of which 53.4% were girls and 46.6% were boys, as shown in Table 1. The students’ ages ranged from 6 to 14 years, and the mean age was (8.06 ± 1.7 years). The majority of the students had completed preschool vaccinations (95.2%). Dental cavities, myopia, and obesity/overweight were the most prevalent health problems for the primary school students, as they were observed in 38.7%, 10.9%, and 10.5% of screened students, respectively (Table 2). ADHD, scoliosis, and auditory health problems were prevalent in only 2.8%, 0.48%, and 0.7% of screened students, respectively.

### ADHD: Attention-Deficit/Hyperactivity Disorder

Health problems that were significantly higher among girls more than boys were scoliosis (0.7% vs. 0.2%), error of refraction (12.1% vs. 9.4%), obesity and overweight (11.3% vs. 9.6%), dental cavities (41.5% vs. 35.6%), and ADHD (1.2% vs. 0.7%). However, incomplete vaccination status and auditory problems were significantly higher among boys than girls (5.2% vs. 4.4% and 1.0% vs. 0.6%, respectively) (*p* = 0.002) (Table 3).

Dental cavities were highly prevalent among primary school students in Jazan (78.1%), Najran (77.9%), Tabouk (75.0%), and Makkah (72.8%). ADHD was prevalent in Al-Baha (51.5%), and obesity and overweight were prevalent in the northern borders (44.7%) and Al-Jouf (38.5%). Errors of refraction were prevalent in the eastern region, Al-Jouf, and Al-Hasa (32.5%, 31.5%, and 30.4%, respectively). The highest prevalence of auditory problems was observed among students in the eastern region (11.7%), while scoliosis prevalence was highest among students in Makkah (1.9%) and the Eastern Region (1.1%). Of all regions, incomplete preschool vaccination was highest among students in Riyadh (24.2%) and Jeddah (19.6%), as shown in Table 4.

## 4. Discussion

This study presents the nationwide prevalence of health problems in school-aged children identified by the screening program. The data presented were collected from first- and fourth-grade Saudi primary school students (*n* = 444,259) during the academic year 2018–2019. This dataset explored variations in the prevalence of the screened conditions across the 20 regions of the KSA. The large sample size and numerous health problems screened for by this program add strength and uniqueness to this study. The prevalence of health problems ranged from 0.48% to 38.7%, which highlights the importance of screening and early detection of health problems, allowing for early management. The proportion of completely immunized for schoolchildren increased from 75% in 1993 to 95.2% in the current study [13,14]. This could be attributed to the efforts of the Saudi MOH in providing childhood vaccinations that are accessible at primary healthcare centers on a daily basis. This effort is further enhanced by MOH endeavors to increase vaccination adherence. MOH, through its vaccination reminder services, contributes to raising awareness and positively affects the attitude of parents towards vaccination, increasing childhood vaccination [15,16]. A recent systematic review conducted in the KSA demonstrated overall positive attitudes and awareness among parents regarding childhood vaccination [17].

Our study showed that the prevalence of overweight and obesity among school students was 6.4% and 4.1%, respectively, which is lower than the rates reported by other local studies, where the prevalence of overweight and obesity was 10.7–14.4% and 7.9–13.9%, respectively [18,19]. Our results are also lower than those reported in the National Growth Study conducted by Al Mouzan et al. in KSA in 2010, where the prevalence of overweight and obesity in schoolchildren aged 5–12 years was 19.6% and 7.9%, respectively [20]. However, several BMI percentile standards are used to define overweight/obesity among the pediatric population, namely the Centers for Disease Control (CDC) standards, World Health Organization (WHO) standards, and country-specific standards [21]. Most previous studies employed the CDC or WHO standards. Moreover, Al-Mendalawi affirmed the possible overestimation of the prevalence of overweight and obesity among Saudi children when using standards other than country-specific growth charts [21]. In 2016, Al Mouzan et al. established a new reference for the evaluation of nutrition- and growth-specific values for Saudi school-age children and adolescents, which provides a more accurate clinical assessment [11]. This chart was used to screen primary school students in the current study. A study conducted in KSA in 2019 found that increasing physical education time in schools was positively associated with an increase in physical activity. The study concluded that Saudi school students did not meet the recommended level of physical activity [22]. This highlights the importance of engaging children in physical education and activity, given its role in reducing the prevalence of overweight and obesity [23].

The prevalence of eye refractory errors (myopia) was 10.9% in our study. This is similar to the findings from a local study, which reported a prevalence of 9% among primary school students aged 6–14 years [24]. However, according to the literature, there are huge variations in the prevalence of myopia among school students. A prevalence of 3.3% was reported among schoolchildren aged 6–7 years old in Ireland [25], 14.9% among 6–15-year-old school students in Iran [26], and 35.8% in 6–8-year-old children in China [27]. Myopia was as high as 63.1–78.6% in 6–8-year-old children in Japan [28]. The discrepancy in the definition of myopia and different screening methods played a significant role in this interstudy variation. Furthermore, in our study, the prevalence of myopia was found to be higher among females (12.1% girls vs. 9.4% boys), which is consistent with other local studies [24]. In contrast, other international studies have found that myopia is higher among boys [26,29].

In the current study, the prevalence of auditory problems was 0.7% among first-grade students. This prevalence is lower than the figures found in international studies, where the prevalence is 8.1% in Canada [30], 19.6% in Brazil [31], and 27.2% in Kyrgyzstan [32]. However, in the latter studies, the screening threshold was 20 dB, while in the current study, it was at 25 dB, resulting in fewer cases detected. Additionally, only students suspected of hearing issues were screened. The assessment was based on the specific signs identified by teachers. Failure to recognize students led to their exclusion from screening which may have resulted in a lower prevalence.

Dental pain and cavities are global issues and may lead to student absences from school and sleep disturbances, hence affecting academic achievement [5]. The prevalence of dental cavities in this study was 38.7%. This is much lower than that reported in a systematic review conducted in KSA in 2013, which reported a prevalence of 70% based on data obtained from children’s primary dentists. However, the review included 26 studies conducted between 1988 and 2010 among different age groups [33], which could explain the variation in the results obtained. It is well known that first permanent molars usually erupt during the first or second grade (6–7 years old), while adolescents at age 13 usually have 28 permanent teeth [34]. Even though the current study included only first-and fourth-grade students, the screening for dental cavities included permanent teeth. Additionally, girls typically erupt permanent teeth much earlier than boys, explaining the higher prevalence of dental cavities in girls compared to boys (41.5% vs. 35.6%) [35,36]. A local study conducted among schoolchildren aged 6–9 years in Riyadh showed that the prevalence of dental cavities was 14.4% [37]. Furthermore, a worldwide systematic review (1995–2019) reported that the prevalence of dental cavities in children’s permanent teeth was 53.8% [38].

The overall prevalence of scoliosis and/or kyphosis was 0.48% among fourth-grade students in this study. This is lower than that reported in two systematic reviews conducted in China and Australia, where the prevalence of scoliosis among students was 1.02% and 1.1%, respectively. Nonetheless, these studies included older children, in whom the prevalence was usually higher [39]. In the present study, the prevalence of scoliosis among girls was higher than that among boys (0.7% vs. 0.2%). This is in line with the results of previous studies that reported that scoliosis was higher among females [40,41]. A study conducted among school boys aged 10–14 years in Al-Kharj reported a prevalence of 0.08% in adolescent idiopathic scoliosis [41], which is lower than the prevalence reported in our study among boys. However, the current study employed a larger sample size, and also included screening for kyphosis.

ADHD is one of the most common mental disorders that usually begins in childhood and becomes more prominent during school years [6]. Worldwide, studies of ADHD among school-aged children revealed a prevalence between 2% and 18% [6]. In KSA, different studies have been conducted and have revealed variations in the overall rates of ADHD, ranging from 2.7% to 7.4% [42,43,44]. This is in agreement with the overall prevalence reported in the current study (2.8%). In contrast to previous studies included in a systematic review of ADHD in Arab countries [43], our study found that ADHD was more prevalent in girls. This finding could be attributed to the teachers’ own judgment in the selection of students suspected of ADHD, which was merely based on signs and symptoms. Anastopoulos et al. reported that the sex of the informant could play a clinically significant role in assessing ADHD in children, as female teachers and parents’ ratings of symptoms were significantly higher than those of male teachers and parents [45].

## 5. Study Limitations

One limitation of this study was the unavailability of sociodemographic data in the school health-screening program. The inclusion of such data could allow for a more meaningful understanding of the determinants of health issues. Another limitation was that screening for ADHD and hearing issues was only carried out in selected students in the first grade, which could obscure the true prevalence of these conditions. Involving all students in both the first and fourth grades could allow for better assessment of the prevalence of ADHD and hearing issues.

## 6. Conclusions

The prevalence of health problems among primary school students ranged from 0.48% to 38.7%. The most common health problems were dental cavities, error of refraction, obesity, and being overweight. These problems were significantly higher among girls than among boys. Appropriate school health programs should be implemented in primary schools in KSA to address and prevent these problems.

## Figures and Tables

**Table 1:** The frequency distribution of students in relation to educational grade and sex.

Parameter	*N* = 444,259	%
Age (Years)		
Mean ± SD	8.06 ± 1.7	
Range	6–14	
Student’s grade		
First grade	219,218	49.3
Fourth grade	225,041	50.7
Sex		
Boys	207,109	46.6
Girls	237,150	53.4

**Table 2:** The prevalence of health problems among screened primary school students in KSA (*N* = 444,259).

Health Problem	*N*	%
Not completed immunization *	10,521	4.80%
Eye refractory errors	48,313	10.90%
Overweight and obesity	46,658	10.50%
Overweight	28,430	6.40%
Obesity	18,228	4.10%
Dental caries	172,113	38.70%
Scoliosis and/or kyphosis **	1086	0.48%
ADHD disorders ***	878	2.81%
Attention deficit (AD)	422	1.35%
Hyperactivity disorder (HD)	165	0.53%
Both (AD plus HD)	291	0.93%
Auditory problems ***	109	0.7%
Right ear	17	0.1%
Left ear	20	0.1%
Both ears	72	0.47%

* Only 1st grade students (*N* = 219,218). ** Only 4th grade students screened (*N* = 225,041). *** Students suspected by parents and teachers only in 1st grade (number of students suspected with ADHD is 31,210, and with auditory problems is 15,426).

**Table 3:** The association of health problems with sex of screened primary school students (*N* = 444,259).

Health Problem	Sex		*p* Value
	Boys	Girls	
	*N* = 207,109	*N* = 237,150	
Incomplete vaccination status *	5354 (5.2%)	5167 (4.4%)	<0.001
Scoliosis and/or kyphosis **	216 (0.2%)	870 (0.7%)	<0.001
Eye refractory errors	19,528 (9.4%)	28,785 (12.1%)	<0.001
Obesity and overweight	19,896 (9.6%)	26,762 (11.3%)	<0.001
Overweight	11,947 (5.8%)	16,483 (7%)	<0.001
Obesity	7949 (3.8%)	10,279 (4.3%)	<0.001
Dental caries	73,701 (35.6%)	98,412 (41.5%)	<0.001
ADHD disorders ***	353 (1.1%)	525 (1.2%)	0.002
Auditory problems ****	52 (1.0%)	57 (0.6%)	0.002

* Only 1st grade students (*N* = 219,218; boys = 102,490, girls = 116,728). ** Only 4th grade students screened (*N* = 225,041; boys =104,619, girls = 120,422). *** Students suspected by parents and teachers only in 1st grade (*N* = 31,210 for ADHD problems; boys, 14,142, girls = 17,068). **** Students suspected by parents and teachers only in 1st grade (*N* = 15,426 for auditory problems; boys = 5186, girls = 10,240).

**Table 4:** Distribution of health problems of screened primary school students according to KSA health regions.

Region	Total Screened	Obesity and Overweight *N* (%)	Errors of Refraction *N* (%)	Dental Caries *N* (%)	Scoliosis *N* * (%)	ADHD *N* ** (%)	Auditory Problems *N* ** (%)	Incomplete Immunization *N* *** (%)
**Riyadh**	68,032	12,725 (18.7)	11,882 (17.5)	42,955 (63.1)	277 (0.4)	160 (1.7)	33 (0.85)	2549 (24.2%)
**Jeddah**	30,291	4384 (14.5)	4007 (13.2)	21,773 (71.9)	77 (0.4)	49 (1.5)	1 (0.1)	2067 (19.6%)
**Eastern Region**	28,133	6724 (23.9)	9139 (32.5)	11,981 (42.6)	212 (1.1)	70 (12.0)	7 (11.7)	986 (9.4%)
**Makkah**	25,841	2781 (10.8)	3927 (15.2)	18,805 (72.8)	326 (1.9)	1 (100.0)	1 (100.0)	953 (9.1%)
**Qassim**	17,913	3168 (17.7)	3641 (20.3)	10,977 (61.3)	29 (0.2)	94 (6.7)	4 (3.5)	670 (6.4%)
**Jazan**	10,953	1303 (11.9)	996 (9.1)	8552 (78.1)	34 (0.3)	54 (2.7)	14 (0.9)	141 (1.3%)
**Hail**	7510	1474 (19.6)	689 (9.2)	5300 (70.6)	14 (0.1)	26 (6.9)	7 (2.7)	344 (3.3%)
**Al-Hasa**	17,510	2868 (16.4)	5320 (30.4)	9061 (51.7)	20 (0.2)	229 (4.9)	12 (0.3)	54 (0.5%)
**Aseer**	8602	1880 (21.9)	1505 (17.5)	5148 (59.8)	19 (0.2)	46 (2.7)	4 (0.3)	443 (4.2%)
**Tabouk**	11,715	1219 (10.4)	1626 (13.9)	8789 (75.0)	5 (0.5)	70 (17.5)	6 (4.8)	920 (8.7%)
**Al-Taif**	6778	927 (13.7)	1081 (15.9)	4702 (69.4)	32 (0.4)	34 (2.6)	2 (1.8)	701 (6.7%)
**Medina**	9787	1593 (16.3)	1115 (11.4)	7070 (72.2)	6 (0.1)	3 (0.5)	0 (0.0)	41 (0.4%)
**Najran**	3568	454 (12.7)	330 (9.2)	2780 (77.9)	3 (0.1)	0 (0.0)	1 (1.9)	262 (2.5%)
**Al-Jouf**	2986	1151 (38.5)	940 (31.5)	889 (29.8)	2 (0.0)	4 (3.4)	0 (0.0)	269 (2.6%)
**Bisha**	2334	518 (22.2)	182 (7.8)	1623 (69.5)	4 (0.2)	4 (0.3)	3 (0.6)	17 (0.2%)
**Northern Borders**	1475	659 (44.7)	89 (6.0)	708 (48.0)	7 (0.2)	3 (0.2)	9 (1.0)	57 (0.5%)
**Hafr Al-Batin**	4190	1052 (25.1)	171 (4.1)	2957 (70.6)	7 (0.2)	0 (0.0)	3 (0.4)	9 (0.1%)
**Al-Baha**	4289	518 (12.1)	769 (17.9)	2981 (69.5)	4 (0.1)	17 (51.5)	0 (0.0)	16 (0.2%)
**Al-Qunfudhah**	4544	661 (14.5)	776 (17.1)	3087 (67.9)	6 (0.2)	14 (3.2)	0 (0.0)	22 (0.2%)
**Al-Qurayyat**	68,032	12,725 (18.8)	11,882 (17.5)	42,955 (63.1)	2 (0.1)	0 (0.0)	2 (100.0)	0 (0.0%)

* Only 4th grade students (*N* = 225,041). ** Students suspected by parents and teachers in each region. *** Only 1st grade students (*N* = 219,218).

## Data Availability

The data presented in this study are available on request from the corresponding author. The data are not publicly available due to privacy concerns.
